# New insights into lactylation in respiratory diseases: progress and perspectives

**DOI:** 10.7717/peerj.20548

**Published:** 2026-01-09

**Authors:** Longmin Chen, Yuan Zou, Qianqian Xu, Jing Zhang

**Affiliations:** 1 Department of Rehabilitation, Tongji Hospital, Tongji Medical College, Huazhong University of Science and Technology, Wuhan, Hubei, China; 2 Department of Respiratory and Critical Care Medicine, Tongji Hospital, Tongji Medical College, Huazhong University of Science and Technology, Wuhan, Hubei, China

**Keywords:** Lactate, Lactylation modification, Molecular mechanism, Respiratory disease, Targeted therapy

## Abstract

Lactate is conventionally regarded as a metabolic byproduct and generated through diverse pathophysiological pathways. However, a growing body of evidence supports its regulatory roles in energy metabolism and signal transduction, boosting extensive research into lactate-mediated lactylation as a newly discovered post-translational modification (PTM). Lactylation can occur on both histone and non-histone proteins, thereby modulating gene transcription and protein function. By influencing various biological processes, lactylation has been shown to intricately participate in the onset and progression of respiratory diseases that are closely related to metabolic abnormalities and remodeling, including asthma, lung cancer, pulmonary fibrosis, silicosis, pulmonary hypertension (PH), and acute lung injury (ALI). In this review, we summarize the current progress in this field, underscoring the multifaceted regulatory and functional mechanisms underlying lactylation, the pivotal role of lactylation in different respiratory diseases, as well with its potential as a therapeutic target. This comprehensive understanding offers novel insights into the pathogenesis of respiratory diseases and opens new avenues for therapeutic approach.

## Introduction

Energy is crucial for maintaining life activities, with glucose being the main energy source that fuels the vitality of all mammals. The production of energy from glucose is primarily facilitated by two interrelated metabolic pathways: oxidative phosphorylation (OXPHOS) and glycolysis (Wang et al., 2024b). Lactate, generated by cells during glycolysis in the absence of oxygen, has traditionally been viewed as a worthless byproduct of metabolism. However, in 1923, Otto Warburg found that tumor cells absorbed glucose more efficiently than normal cells and continued to favor glycolysis even when oxygen was available, leading to high lactate production and energy generation. This phenomenon is termed the Warburg effect or aerobic glycolysis, and lactate serves as a metabolic substrate that supports the proliferation of cancer cells (Vander Heiden, Cantley & Thompson, 2009). With advancements in our understanding of cellular metabolism, it has become evident that aerobic glycolysis is not exclusive to cancer cells. It is also observed in noncancerous diseases, such as tuberculosis, pulmonary hypertension, idiopathic pulmonary fibrosis (IPF), atherosclerosis, cardiac hypertrophy, heart failure, multiple sclerosis, Alzheimer’s diseases, and polycystic kidney disease (Chen et al., 2018), highlighting the important role of lactate in the pathophysiological processes underlying these clinical conditions. In recent years, researchers develop the lactate shuttle theory. This concept depicts that lactate can not only shuttle intracellularly or between producer and consumer cells for delivery of oxidative and gluconeogenic substrates, but also act as a crucial signaling molecule with autocrine-, paracrine- and endocrine-like effects (Brooks, 2018). As a multifaceted signaling molecule, lactate is capable of regulating fibrosis, angiogenesis, and immune response, but the detailed regulatory mechanisms still need further study (Certo et al., 2022).

Lysine lactylation (Kla), a new type of post-translational modification (PTM) of proteins, was first reported by Zhang et al. (2019). By employing peptide immunoprecipitation combined with high-performance liquid chromatography (HPLC)-tandem mass spectrometry (MS/MS) analysis, they identified 26 and 16 lactylation sites on histone lysine residues in human Hela cells and mouse bone marrow-derived macrophages (BMDMs), respectively, which directly stimulated gene transcription from chromatin (Zhang et al., 2019). Moreover, the research team found that glycolytic inhibitors decreased levels of both intracellular lactate and histone Kla, whereas mitochondrial inhibitors or hypoxia exposure that drove cells towards glycolysis increased both lactate production and histone Kla levels (Zhang et al., 2019). The discovery of histone lactylation thus improves our understanding of the functional implications of the Warburg effect, as lactate is a key determinant for this modification. Subsequent research has shown that lactylation occurs not only on histones, but also on a variety of non-histone proteins, particularly oncoproteins and enzymes involved in metabolic pathways (Yang et al., 2023), and exerts its functions in multiple physiological and pathological contexts (Li et al., 2024b).

Respiratory diseases are a group of conditions that affect the airways and other structures of the lungs, leading to difficulty breathing and various symptoms. Common causes of respiratory diseases include infections, tobacco smoke, air pollution, allergens, occupational exposures, and genetic factors. Respiratory diseases are among the leading causes of morbidity and mortality worldwide, with five conditions that primarily account for the global burden of respiratory disease: chronic obstructive pulmonary disease (COPD), asthma, acute respiratory infections, tuberculosis (TB), and lung cancer (Ferkol & Schraufnagel, 2014; Zheng et al., 2023). Particularly, the chronic respiratory diseases (CRDs) are the third leading cause of mortality in 2019, responsible for 4.0 million deaths with a prevalence of 454.6 million cases globally (Collaborators, 2023). In order to promote respiratory health and reduce the social and economic burden, research into uncovering molecular mechanisms of these diseases and developing new treatments are important. Emerging evidence has established the intricate relationship between lactylation and respiratory diseases. Here, we provide an overview of lactate homeostasis, introduce the dynamic process of lactylation, and comprehensively review its involvement in certain respiratory diseases. Moreover, we discuss potential targets and candidate intervention agents for lactylation, offering new perspectives on disease prevention and treatment. This review is intend for both basic scientists and clinicians in the related field.

## Survey Methodology

Several pivotal steps were taken to guarantee the comprehensiveness and accuracy of this review (Fig. S1). First, a literature search on PubMed and Web of Science was performed from inception to 2025. The search terms used included “lactate”, “lactylation”, “respiratory disease”, “asthma”, “lung cancer”, “pulmonary fibrosis”, “silicosis”, “pulmonary hypertension”, “acute lung injury” and “therapeutic target”. These terms were combined using Boolean operators AND/OR. Following database retrieval, we removed duplicate articles and implemented a two-stage screening protocol: initial triage on the basis of title/abstract relevance, followed by a further full-text evaluation using predefined criteria: (1) inclusion of original research articles, reviews, letters, and clinical trials published in English; exclusion of editorials, viewpoint, and conference abstracts; (2) methodological rigor, *e.g.*, controlled experiments and adequate sample size; (3) prioritization of high-impact studies. In addition, we searched the reference lists of included articles to identify relevant publications, thereby maintaining the integrity of our review.

## Lactate Homeostasis

### Lactate production and clearance

**Figure 1:**
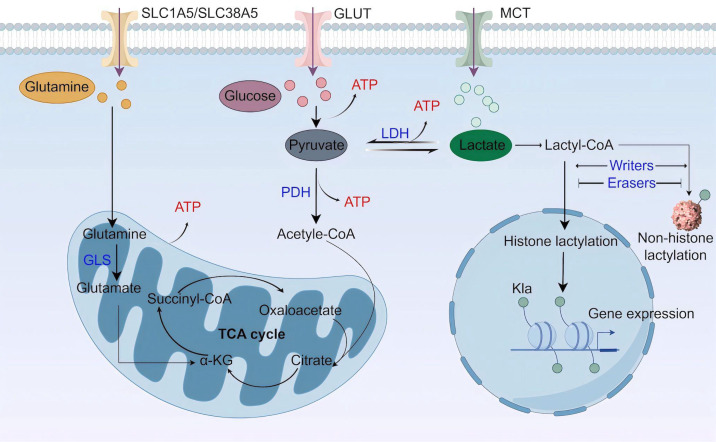
Lactate metabolism and lactylation. MCT, monocarboxylate transporter; LDH, lactate dehydrogenase; PDH, pyruvate dehydrogenase; GLS, glutaminase; TCA cycle, tricarboxylic acid cycle (Figure was created with figdraw.com).

Lactate is a best-known byproduct of glucose catabolism, and mainly generated through the glycolytic pathway (Fig. 1). Glycolysis and lactate production are increased when the demand for oxygen and adenosine triphosphate (ATP) exceeds supply, as seen in high-intensity exercise, ischaemia, and septic shock (Rabinowitz & Enerback, 2020). When glycolysis occurs, one glucose molecule is converted into two pyruvate molecules through a series of enzyme-catalyzed reactions in the cytoplasm. Along with pyruvate, the process also yields two ATP, two reduced form of nicotinamide adenine dinucleotide (NADH), and two H_2_O. Pyruvate staying in the cytoplasm can be subsequently converted to lactate by the enzyme lactate dehydrogenase (LDH) (Gray, Tompkins & Taylor, 2014). Besides glycolysis, glutaminolysis is another source of lactate in tumor cells (De Berardinis et al., 2007). Glutamine transports across cell membranes through the transporters SLC1A5 (solutecarrier family 1 member 5; also known as ASCT2) and SLC38A5 (solutecarrier family 38 member 5; also known as SN2) (Shen et al., 2021), enters the mitochondria *via* the SLC1A5 variant, a mitochondrial glutamine transporter (Yoo et al., 2020a), and is converted to glutamate by glutaminase (GLS). Next, mitochondrial glutamate is converted to alpha ketoglutarate (α-KG), which can participate in the tricarboxylic acid (TCA) cycle (Yoo et al., 2020b). In the TCA cycle, glutamine-derived carbon is converted to malate, which then enters the cytoplasm for subsequent conversion to NADPH and pyruvate. NADPH supports redox homeostasis and is utilized to synthesize fatty acids (FAs) and cholesterol, while pyruvate is a source for lactate production.

Lactate buildup in the human body poses a greater risk compared to other molecular fuels, and an increase in serum lactate can result in lactic acidosis (Bennis et al., 2020). Thus, lactate needs to be swiftly cleared. The elimination of intracellular lactate is achieved by pyruvate dehydrogenase (PDH), which catalyzes the irreversible conversion of pyruvate to acetyl coenzyme A (acetyl-CoA) (Jha, Lee & Suk, 2016). Acetyl-CoA then enters the mitochondrial TCA cycle. In addition, lactate can be transferred to the liver and skeletal muscle, where it undergoes gluconeogenesis in a process called Cori cycle or lactic acid cycle. The newly synthesized glucose is subsequently released into the bloodstream, providing a continuous supply of energy (Emhoff et al., 2013). A recent study highlights the mitochondrial lactate oxidation complex (mLOC) as a lactate oxidation catalyst (Brooks et al., 2022). The mLOC is organized on the inner mitochondrial membrane, comprising several essential components: a mitochondrial monocarboxylate transporter (mMCT), its chaperone protein CD147, mitochondrial LDH (mLDH), and cytochrome oxidase (COx) (Brooks, 2018). Mitochondrial LDH oxidizes cytosolic lactate to pyruvate, which would be transported into the mitochondria *via* mMCT and eventually oxidized in the TCA cycle. Nevertheless, most studies on lactate oxidation concentrate on the LDH-mediated axis in oxidative cells, and more work on issues related to the role of mLOC in pathophysiological processes is required.

### Lactate shuttle and signaling

Lactate can be transported across plasma membranes in a MCTs-dependent manner. The transport direction depends on the lactate gradient and is from areas of high concentration to those of lower concentration. Structurally, MCTs are predicted to contain 12 transmembrane helices (TMs) with a long intracellular loop between TM6 and TM7, as well as the N- and C-termini (Jones & Morris, 2016). At present, 14 MCT family members have been identified, among which MCT1-4 are extensively studied and responsible for unidirectional proton-linked transport of monocarboxylates (Halestrap, 2013; Pucino, Cucchi & Mauro, 2018). These transporters have distinct expression pattern and affinity for lactate (Llibre et al., 2021). While MCT1 is widely expressed, MCT2 is predominantly found in the liver, kidney, testis and brain. MCT2 has the highest lactate affinity, and both MCT1 and MCT2 mediate the uptake of lactate into cells. MCT3 is restricted to retinal pigment and choroid plexus epithelia, where it serves as a lactate exporter. MCT4 is strongly expressed in tissues with a high glycolytic rate, has a low affinity for lactate and is responsible for lactate export (Bosshart et al., 2019).

Classical metabolites can signal directly *via* G-protein-coupled receptors (GPCRs) (Husted et al., 2017). Specifically, lactate can trigger a signaling pathway though its receptors GPR81 and GPR132, both being proton-sensing GPCRs (Certo et al., 2022; Baltazar et al., 2020). GPR81 is mainly expressed in adipose tissue, also found in immune cells, the central nervous system, muscle cells, and now tumor cells (Brown & Ganapathy, 2020). Studies have shown that lactate signaling through GPR81 has multiple effects, such as inducing lipid accumulation (Cai et al., 2008; Liu et al., 2009), favoring tumor growth (Yang et al., 2020b; Qu et al., 2021; Feng et al., 2017; Wagner et al., 2017), inflammatory regulation (Yang et al., 2020a; Madaan et al., 2017), and neuronal protection (Buscemi et al., 2021; Harun-Or-Rashid & Inman, 2018). GPR132 is expressed in the lung, gastrointestinal tract, and immune cells, especially macrophages. Current studies on lactate-GPR132 signaling are limited and mostly conducted in mice. However, the murine and human GPR132 have different proton sensitivity (Radu et al., 2005) and only about 67% overall identity in the amino acid sequence (Obinata & Izumi, 2009).

## Overview of Lactylation

Kla, a newly identified PTM, is derived from intracellular accumulation of lactate. Kla has been verified to occur on both histones and non-histone proteins. Given that lactate is a chiral compound, Kla exists in two distinct forms: enzymatic lactylation (lysine L-lactylation, K_L-la_) and non-enzymatic lactylation (lysine D-lactylation, K_D-la_) (Liao et al., 2025). However, the primary lactylation isomer is the L form rather than the D form *in vivo*, because K_D-la_ is only observed when the glyoxalase pathway is incomplete (Zhang et al., 2025a). Therefore, the term “lactylation” exclusively refers to L-lactylation hereafter.

### Histone lactylation

Since lactate-derived lactylation was first identified on histone lysine residues by Zhao’s research group (Zhang et al., 2019), a growing number of histone Kla sites have been recognized. The researchers find that histone lactylation occurs on core histones H3, H4, H2A, and H2B, especially lysine 18 on H3 (H3K18). H3K18 lactylation predominantly locates at gene promoters and tissue-specific active enhancers, and is positively correlates with H3K4me3 and H3K27ac as well as with gene expression (Galle et al., 2022). H3K18la is associated with a variety of biological processes, including tumorigenesis, macrophage polarization, gene transcription regulation, and stem cell and embryonic development (Galle et al., 2022). In addition to H3K18, lactylation-related sequencing has discovered other histone Kla sites, such as H2AK11, H2BK6, H2BK16, H2BK120, H3K9, H3K14, H3K23, H3K56, H4K5, H4K12, H4K16, and H4K80, but the specific functions performed by these modifications require further investigation (Jing et al., 2025; Zhao et al., 2025). Moreover, given that the majority of previous researches only focus on specific gene sets, whether histone lactylation generally promotes or suppresses gene transcription remains to be determined, which might be context-dependent. The use of combinatorial methodologies, such as histone lactylation ChIP-seq coupled with paired RNA-seq, should help to clarify the general association between histone lactylation and gene transcription in an unbiased manner.

### Lactylation of non-histone proteins

In recent years, studies have shown that Kla modifications extend to non-histone proteins, thereby regulating protein structure, localization, stability, interactions, or functions by either enhancing or inhibiting the original functions of these proteins. LC-MS/MS analysis of protein lactylation levels in the spleen, muscle, liver and heart of healthy rats identifies 1,027 Kla sites on 433 proteins, and thereinto histone lactylation makes up less than 3% (Lin et al., 2022). The occurrence of protein lactylation is widespread, appearing in the nucleus, cytoplasm, and multiple organelles. However, Kla-modified proteins in the nucleus account for no more than 17%, encompassing all histone lactylation, while more lactylated proteins are distributed in the cytoplasm (Lin et al., 2022). In patients with hepatitis B virus (HBV)-related hepatocellular carcinoma (HCC), global lactylome and proteome analyses identify a total of 9,275 Kla sites from all 110 samples, with 9,256 sites on non-histone proteins. Moreover, the lactylated proteins are more represented in the cytosol (Yang et al., 2023). Two other studies focus on retinal neovascularization and heart failure, in which lactylome analyses of microglia under hypoxia and myocardial tissues from heart failure mice detect 3,093 lactylation sites on 751 proteins (Wang et al., 2023b) and 551 lactylation sites on 160 proteins (Zhang et al., 2023b), respectively. These results imply that lactylation is a common modification beyond histones and has a broader biological role than transcriptional regulation.

### The substrates and enzymes involved in lactylation

The lactylation process is dynamically regulated by specific enzymes or enzyme complexes classified as “writers”, “erasers”, and “readers”. Lactyltransferases, referred to as “writers”, catalyze the addition of lactyl groups to lysine residues, while delactylases, known as “erasers”, remove these lactyl groups. “Readers” are responsible for recognizing Kla sites, and bring about distinct downstream events. For PTMs originated from internal metabolites, there are also pertinent enzymes that convert them into direct PTM donors. Collectively, these enzymes offer an elaborate mechanism for the fine-tuning of lactylation, contributing to its function as a key biological regulator.

Lactyl-CoA serves as a substrate for Kla, forming a reversible covalent bond with the lysine residues of target proteins. Acyl-CoA synthetases (ACSs) are a family of enzymes that mediate the thioesterification of FAs with CoA to form activated intermediates, which have an important role in FA metabolism (Black & Di Russo, 2007). Due to structural similarities between the lactyl group and the FA group, it is speculated that ACS family enzymes may be involved in the conversion of lactate into lactyl-CoA. This idea is supported by a recent study, in which guanosine triphosphate (GTP)-specific SCS (GTPSCS) was identified as a lactyl-CoA synthetase from 12 selected fatty acyl-CoA synthetases (Liu et al., 2025a). In the nucleus, GTPSCS utilizes the lactate generated by tumors and produces lactyl-CoA for *in situ* H3K18la, which in turn support glioma proliferation and radioresistance (Liu et al., 2025a). As an inaugural lactyl-CoA synthetase, additional investigation is required to decipher the role of GTPSCS in lactylation across a range of physiological and pathological conditions.

Lysine acetylase p300 is the first identified histone Kla writer protein (Zhang et al., 2019). Other studies have also reported that both p300 and its homologue CREB binding protein (CBP) are involved in lactate-promoted high mobility group box-1 (HMGB1) lactylation in macrophages (Yang et al., 2022). In *Escherichia coli*, YdiF and YiaC catalyze the formation of lactyl-CoA and addition of Kla, respectively, promoting bacterial glycolysis and growth (Dong et al., 2022). HBO1, a versatile histone acyltransferase, has been reported to catalyze lysine lactylation and mediate histone H3K9la to regulate gene transcription (Niu et al., 2024). In addition, the lysine acetyltransferases KAT5 and KAT8 are discovered to install Kla on many non-histone substrates (Jia et al., 2023; Xie et al., 2024), while the alanyl-tRNA synthetases AARS1 and AARS2 act as global lysine lactyltransferases, directly catalyzing L-lactate for ATP-dependent lactylation (Li et al., 2024a). On the other hand, class I histone deacetylases (HDAC1-3) and SIRT1-3 are well-defined “erasers” of lactylation. HDAC1-3 are the main histone lysine delactylases (Moreno-Yruela et al., 2022), whereas SIRT1 and SIRT3 are robust lysine delactylases of both histone and non-histone Kla (Du et al., 2024). Intriguingly, there is minimal overlap between SIRT1 and SIRT3-targeted Kla sites, indicating different regulatory patterns and substrate specificities of these two enzymes toward Kla (Du et al., 2024). A proteomic study by Hu et al. (2024) revealed that the bromine-containing protein Brg1 interacted with H3K18la, acting as a reader of histone lactylation to facilitate the process of induced pluripotent stem cell (iPSC) reprogramming. However, the study of Kla-specific readers is still in its early stage, probably because lactylation is only recently discovered and the molecular mechanisms are not yet fully understood.

## Role of Lactylation in Respiratory Diseases

**Figure 2:**
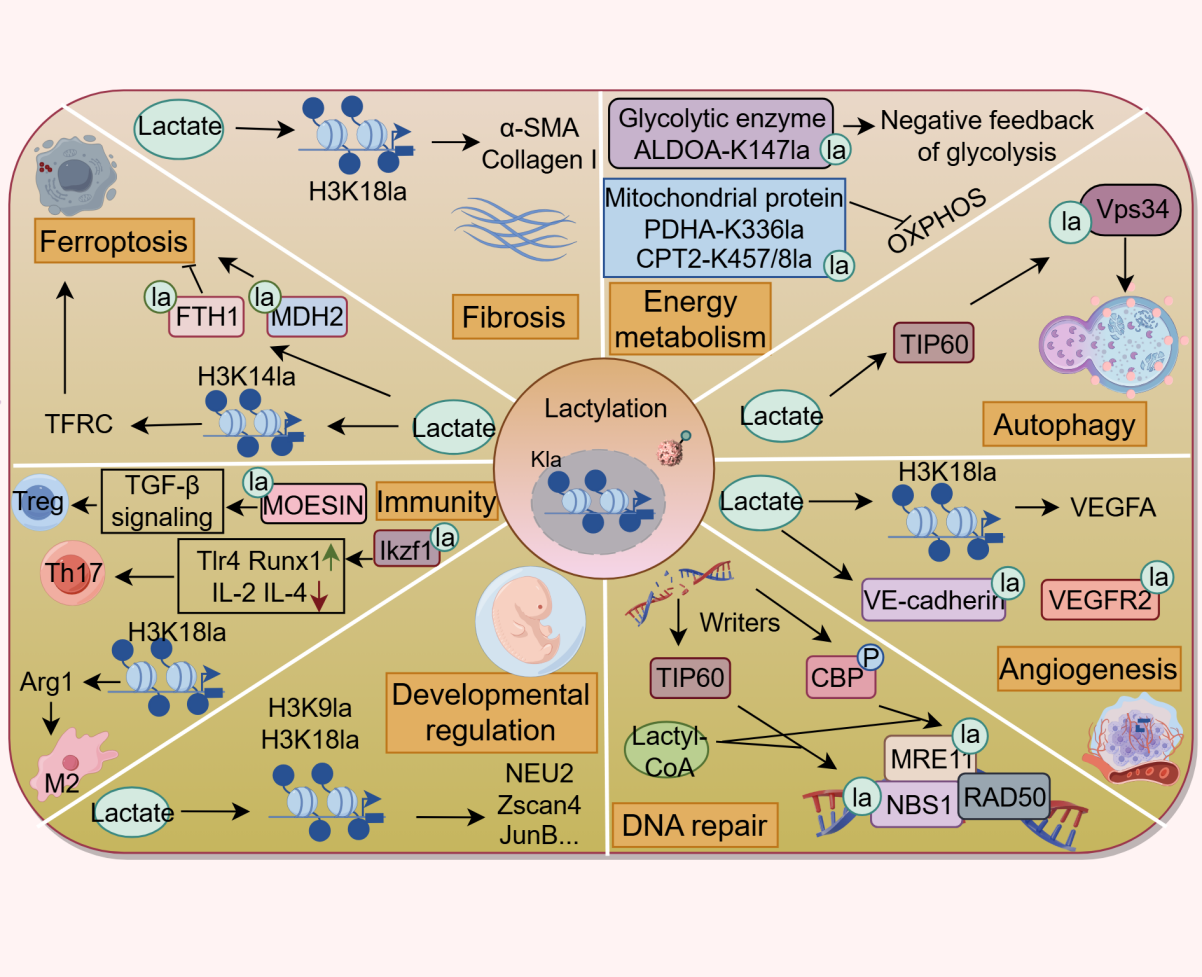
Lactylation contributes to various pathophysiological processes. Lactylation is involved in the regulation of energy metabolism, autophagy, ferroptosis, DNA repair, fibrosis, developmental regulation, angiogenesis, and immune response. (Figure was created with figdraw.com).

**Figure 3:**
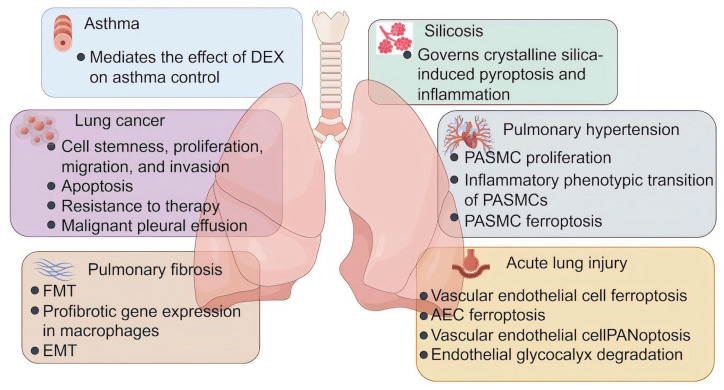
The role of lactylation in respiratory diseases. Lactate plays an important role in the development and progression of asthma, lung cancer, pulmonary fibrosis, silicosis, pulmonary fibrosis, and acute lung injury. DEX, dexamethasone; FMT, fibroblast to myofibroblast transition; EMT, epithelial-mesenchymal transition; PASMC, pulmonary artery smooth muscle cells; AEC, alveolar epithelial cells (Figure was created with figdraw.com).

In the past years, the protein Kla has attracted considerable interest for its profound influences on multiple biological and pathological processes, such as energy metabolism, autophagy, angiogenesis, fibrosis, ferroptosis, and immune responses (Fig. 2). All of these are closely related to the onset and progression of various human diseases. This section discusses the functional implications of Kla across diverse respiratory disorders (Fig. 3 and Table S1), shedding light on the contribution of lactate to respiratory disease pathogenesis from a PTM perspective.

### Asthma

Asthma is a heterogeneous condition marked by chronic airway inflammation and remodeling that lead to different symptoms, treatment responses, and natural history throughout the life of the patients (Holgate et al., 2015). It is estimated that the prevalence of asthma around the world is approximately 10% in children and adolescents and 6–7% in adults, affecting around 300 million people (Porsbjerg et al., 2023). Although the death rate for asthma shows a consistent decline from 2001 to 2015, it is still responsible for over 420,000 deaths each year (Hashmi & Cataletto, 2025). To prevent the avoidable motility of asthma, doctors are called upon to ensure that every person with asthma is prescribed evidence-based, essential, inhaled corticosteroids (ICSs) in combination with reliever medication.

As the mainstay asthma therapy, there is a general belief that corticosteroids prevent asthma attacks by treating the underlying inflammation. Meanwhile, corticosteroids have also been implicated in the regulation of cellular energy metabolism. For example, dexamethasone, the most commonly prescribed corticosteroid, is demonstrated to decrease the basal glycolytic flux of rat hepatocytes (Probst & Jungermann, 1983), suppress glycolysis and enhance mitochondrion function of acute lymphoblastic leukemia cells (Aoki et al., 2017), and reduce glycolysis, glutaminolysis, and fatty acid synthesis in the lung following *Aspergillus fumigatus* exposure (Gan et al., 2023). As metabolic reprogramming towards glycolysis plays an important role in the pathogenesis of asthma (Van de Wetering et al., 2020; Qian et al., 2018; Sha et al., 2023), Chen et al. (2024b) explore whether corticosteroids modulate glycolysis and subsequent protein lactylation in murine models of eosinophilic asthma. They find that dexamethasone downregulates OVA-induced Hif-1α-glycolysis axis in lung macrophages during asthma, leading to lower levels of serum lactate. A similar effect also exists in human macrophage cell line THP-1 (Chen et al., 2024b). Most importantly, OVA challenge remarkably induces protein lactylation in the mouse lung tissues and THP-1 cells, which are both attenuated by dexamethasone (Chen et al., 2024b). This suggests that the effect of dexamethasone on asthma management is related to its inhibition of Hif-1α-glycolysis-lactate axis and resultant lactylation modification. However, additional studies that identify lactylated substrates and Kla sites specifically implicated in asthma are needed.

### Lung cancer

Lung cancer is a very aggressive and highly prevalent disease, causing a significant public health concern (Leiter, Veluswamy & Wisnivesky, 2023). GLOBOCAN 2022 estimates of cancer incidence and mortality indicate that lung cancer is the most frequently diagnosed cancer in 2022, responsible for almost 2.5 million new cases, and the leading cause of cancer-related deaths worldwide, with an estimated 1.8 million deaths (Bray et al., 2024). Based on the morphological features, lung cancer can be classified into non-small cell carcinoma (NSCLC) and small cell carcinoma (SCLC). NSCLC comprises > 85% of total diagnosis and can be further categorized into adenocarcinoma, squamous cell carcinoma, and large cell carcinoma.

As described above, cancer cells utilize an altered metabolic pattern compared to that of normal cells in the body. The cancer cells take up much more glucose and mainly process it through glycolysis regardless of the oxygen levels, with a reduced use of OXPHOS (Levine & Puzio-Kuter, 2010). This metabolic reprogramming results in the production of large amounts of lactate, which may profoundly impact tumor progression and treatment response (Yu et al., 2025). The discovery of lactylation reveals a novel lactate-related mechanism and opens new avenues for developing innovative therapeutic strategies. Lactate is found to modulate cell metabolism (Jiang et al., 2021), suppress ferroptosis (Wu et al., 2025), and potentiate immune escape (Zhang et al., 2024a) in NSCLC cells, and these effects are principally through histone lactylation-mediated expression of related genes. In a xenograft mouse model, inhibition of histone lactylation using glycolysis inhibitor 2-DG or LDHA inhibitor Oxamate can suppress the malignant progression of lung adenocarcinoma (LUAD) (Wang et al., 2023a). Mechanistically, lactate produced by glycolysis significantly increases H3K18la enrichment on the *Idh3g* promoter and its expression, thereby promoting LUAD cell proliferation, migration, and invasion, while inhibiting apoptosis (Wang et al., 2023a). Results from another experimental study of LUAD show that H4K8 and H4K16 lactylation facilitates Sp1 transcriptional activity. Telomerase reverse transcriptase (TERT), a core subunit of telomerase, is a direct target downstream of Sp1. Ultimately, Sp1-mediated TERT transcription maintains telomere length in LUAD cells, allowing them to proliferate without being arrested by replicative senescence (Liu et al., 2024b). Furthermore, hypoxia induces lactylation modification of SOX9 to enhance the stemness, migration, and invasion of NSCLC cells by promoting glycolysis (Yan et al., 2024), suggesting that lactylation of non-histone proteins is also implicated in cancer progression.

Current treatments for lung cancer include surgery, radiotherapy, chemotherapy, targeted therapy, and immunotherapy (Ashrafi et al., 2022). Despite advances in therapeutic options, the problem of resistance to therapy remains the principal limiting factor to achieve cures in patients with cancer (Vasan, Baselga & Hyman, 2019). Elucidating mechanisms of resistance might help improve drug response and treatment outcomes. Evidence suggests that cell plasticity and neuroendocrine differentiation in LUAD are one of the major reasons for therapeutic resistance to targeted therapy such as EGFR inhibitors (Oser et al., 2015), but the contribution of metabolic changes to this phenomenon is not yet fully understood. One experimental study finds that dysfunction of Numb/Parkin-mediated mitochondrial clearance in LUAD leads to an altered metabolic program featured with a significant increase in lactate production, which causes transcriptional surges of neuroendocrine-associated genes *via* the regulation of histone lactylation (He et al., 2023). In brain metastatic lung cancer cells, H4K12la promotes transcriptional activation of *CCNB1* and accelerates the DNA replication and cell cycle, resulting in the chemotherapeutic resistance to pemetrexed (PEM) (Duan et al., 2023). In addition, global lactylome profiling and metabolomic analyses of NSCLC patient samples reveal that lactate induces APOC2 lactylation at K70, stabilizing the protein levels by inhibiting its ubiquitination. This leads to free fatty acid (FFA) release, regulatory T (Treg) cell accumulation, immunotherapy resistance, and tumor metastasis (Chen et al., 2024a). These findings are further validated *in vivo* using the APOC2^K70-lac^ neutralizing antibody in combination with anti-PD-1 therapy, which shows a significant reduction in immunotherapy resistance (Chen et al., 2024a).

Lung cancer can cause fluid to accumulate in the chest, called malignant pleural effusion (MPE). MPE represents a continuous hypoxic environment, where tumor cells and immune cells exhibit the general upregulation of glycolytic pathways and produce significant amounts of lactate (Huang et al., 2021). Lactate modulates the gene transcription of NF-κB p65 through H3K18la, subsequently upregulating TNFR2 expression on Treg cells in MPE. TNFR2^hi^ Treg cells display elevated levels of immune checkpoint molecules and exert more potent immunosuppressive function, thereby promoting the progression of MPE. Notably, targeting lactate metabolism effectively potentiates response to anti-PD-1 therapy in the MPE mouse model of NSCLC, and ameliorates immunosuppression in the MPE microenvironment (Xue et al., 2024). Meanwhile, results from the same research team find that similar to Treg cells, FOXP3^+^ natural killer T (NKT)-like cells from MPE patients highly express MCT1 and LDHB to take in and utilize lactate, thereby maintaining their immunosuppressive function and high H3K18la levels (Wang et al., 2023c). Moreover, MCT1 inhibitor 7ACC2 substantially inhibits the expression of FOXP3 and histone Kla levels in NKT-like cells *in vitro* (Wang et al., 2023c). These findings provide novel insights into MPE treatment from a metabolic perspective.

### Pulmonary fibrosis

Pulmonary fibrosis is a condition characterized by inflammation and progressive lung scarring, resulting in decreased lung compliance, compromised gas exchange, shortness of breath, and ultimately respiratory failure and death (Adegunsoye, 2023; Fainberg et al., 2024). IPF is the most common and severe type of pulmonary fibrosis, with a median survival of 3–5 years from diagnosis (Adegunsoye, 2023). IPF is now thought to result from the interaction of genetic and environmental factors, with recurrent injury to the alveolar epithelial cells (AECs) superimposed on accelerated epithelial aging playing a central role (Lederer & Martinez, 2018). A dysregulated repair of the injured alveolus triggers abnormal epithelial-fibroblast communication, the induction of matrix-producing myofibroblasts, extracellular matrix (ECM) deposition, and the eventual remodeling of lung interstitium (Richeldi, Collard & Jones, 2017; Sankari, Chapman & Ullah, 2025).

The past decade has seen an increasing recognition of the importance of metabolic dysregulation in the pathogenesis of fibrosis. One of the most prominent characteristics of pulmonary fibrosis are mitochondrial dysfunction and glycolytic reprogramming in multiple cells including AECs, macrophages, fibroblasts, and others, which promote the fibrotic response to injury (Yan et al., 2023; Bueno et al., 2020). Fibroblast to myofibroblast transition (FMT) is considered to be the main source of myofibroblasts in fibrotic lungs (Zhang et al., 2023a), and enhanced aerobic glycolysis serves as a crucial step in this process and causes an increase in the levels of lactate (Xie et al., 2015). Consistent with the role of lactate in promoting lactylation, levels of Pan-Kla, H3K18la and H4K12la are increased in both lung tissues from silica-induced pulmonary fibrotic mice and TGF-β1 + IL-1β-stimulated fibroblasts (Liu et al., 2024a). H3K18la enrichment at the promoters of profibrotic genes *Acta2* and *Col1a1* induces mRNA transcription of α-SMA and collagen I, contributing to fibroblast activation and differentiation (Liu et al., 2024a). In another experimental study, extracellular lactate generated by myofibroblasts is transported into AECs through MCT1, which upregulates global lactylation and H3K18la levels, thereby driving the transcription of gene encoding m^6^A reader YTHDF1. YTHDF1 promotes the translation of *Nrep* by recognizing its m^6^A sites, which stimulates the secretion of TGF-β1, and further facilitates FMT and the progression of arsenite-related IPF (Wang et al., 2024a). Myofibroblast-derived lactate has also been reported to induce histone lactylation at the promoters of the profibrotic genes in macrophages (Cui et al., 2021). This effect is mediated by p300, and aids in our understanding of the mechanistic contribution of myofibroblast glycolysis to pulmonary fibrosis development. Epithelial-mesenchymal transition (EMT) represents an early and essential event in the pathogenesis of pulmonary fibrosis (Chapman, 2011). In mice with PM2.5-associated pulmonary fibrosis, PM2.5-induced glycolysis and subsequent histone lactylation increase the expression of profibrotic genes in macrophages, such as *Tgfb*, *Vegfa*, and *Pdgfa*. The secretion of profibrotic mediators from macrophages promotes the process of EMT in lung epithelial cells, resulting in pulmonary fibrosis (Li et al., 2024c). Additionally, it is now apparent that lactylation extensively participates in the pathological progression of many other fibrotic diseases, such as kidney fibrosis (Wang et al., 2024c), cardiac fibrosis (Fan et al., 2023), and liver fibrosis (Zhou et al., 2024; Rho et al., 2023). Given that drug development in the fibrosis field remains limited, there is an urge need for conversion of these knowledge from the bench to the bedside.

### Silicosis

Silicosis refers to an occupational lung disease caused by inhaling very tiny crystalline particles of silicon dioxide, or silica, with a profound impact on workers in industries where exposure to silica dust is prevalent (Leung, Yu & Chen, 2012). The pathophysiology of silicosis involves the phagocytosis of crystalline silica and the subsequent inflammatory and fibrotic responses in the lungs. When silica dust is inhaled, the fine particles reach the terminal bronchioles and alveoli, where they are engulfed by alveolar macrophages. However, silica is toxic to these cells, which are damaged and die, releasing inflammatory substances that attract more macrophages and other inflammatory cells to the site. The ongoing cycle of inflammation and cell death causes the formation of silicotic nodules, the classic histopathological finding of silicosis. With the progression of the disease, normal lung tissue is replaced by fibrotic tissue, leading to scarring and stiffening of the lungs. Recent researches have reported that in the mouse silicosis model, crystalline silica-induced lung inflammation is accompanied by glycolytic reprogramming and pyroptosis of alveolar macrophages, and suppressing glycolysis mitigates crystalline silica-induced inflammatory response by inhibiting NLRP3 inflammasome-dependent pyroptosis (You et al., 2024). Moreover, the authors observe elevated levels of histone lactylation in crystalline silica-treated macrophages, while glycolysis inhibition and lactate blockade reduce lactylation levels, as well as NLRP3 inflammasome activation (You et al., 2024). These findings suggest that increased lactate and lactylation might represent significant mechanisms in crystalline silica-induced NLRP3-dependent macrophage pyroptosis, but the specific targets that mediate inflammasome activation need to be pinpointed by further screening and verification.

### Pulmonary hypertension

Pulmonary hypertension (PH) encompasses a heterogeneous group of disorders characterized by elevated pulmonary artery pressures that can subsequently culminate in right heart failure and premature death (Oldroyd, Manek & Bhardwaj, 2025; Pullamsetti et al., 2020). It is estimated to affect approximately 1% of the global population and can occur in individuals of any age (Kovacs et al., 2024). PH is associated with adverse pulmonary vascular remodeling, mainly manifested by excessive proliferation of pulmonary artery smooth muscle cells (PASMCs) (Mocumbi et al., 2024; Humbert et al., 2019). A plethora of molecular signaling abnormalities have been incriminated in the pathogenesis of PH, among which hypoxia-inducible factor (HIF) signaling has garnered significant attention (Pullamsetti et al., 2020). Chen et al. (2023) find that HIF-1α shifts energy metabolism from OXPHOS to glycolysis in PASMCs, leading to lactate accumulation and histone lactylation. The enhanced H3K18la modification of proliferation-related genes, such as *Bmp5*, *Kit*, and *Trpc5*, boosts the PASMC proliferative phenotype, which facilities pulmonary vascular remodeling in PH rat (Chen et al., 2023). In addition, pharmacological intervention with LDH inhibitor ameliorates vascular remodeling and PH by reducing lactylation modification, providing a proof of concept for anti-proliferative therapy *via* manipulation of lactate. Furthermore, two other researches also confirm that histone lactylation transcriptionally enhances gene expression that contribute to PASMC proliferation and pulmonary vascular remodeling in hypoxia-induced PH rats (Chen et al., 2025; Zhang et al., 2025b). Under hypoxic condition, lactate induces H3K18la at the promoters of *Il1b*, *Il6*, and *tnf*, leading to the transition of PASMCs into the inflammatory phenotype (Zhu et al., 2025). Inflammatory phenotypic transition of PASMCs is a major pathological change in pulmonary artery remodeling, which is followed by the development of PH. More recently, ca-circSCN8A, a chromatin-associated RNA, is found to be upregulated in PH (Li et al., 2025). It recruits EP300 to catalyze FUS lactylation, thereby driving the formation of a ca-circSCN8A/ EP300/FUS complex through liquid-liquid phase separation (LLPS). This process enables ca-circSCN8A to form an R-loop with the promoter of *SLC7A11* gene and inhibits its transcription, resulting in the disruption of the redox homeostasis and hypoxia-induced ferroptosis in human PASMCs (Li et al., 2025). Given the important role of ferroptosis in PH pathogenesis, future studies should designed to validate the significance of the ca-circSCN8A/EP300/FUS/SLC7A11 axis *in vivo* using animal models.

### Acute lung injury

Acute lung injury (ALI) is a common and devastating complication arising early in the course of sepsis, the development of which is associated with high mortality rates in septic patients (Genga & Russell, 2017). The progression of ALI involves three stages: exudative, proliferative and fibrotic stages (Dong et al., 2024; Guan et al., 2023). Interstitial and alveolar edema are key pathological features in the exudative stage (Matthay et al., 2019). Elevated permeability to protein and liquid across the lung endothelium results in edema in the lung interstitium. Then the injured tight barrier properties of the alveolar epithelium facilitate the translocation of the protein-rich inflammatory edematous fluid to the alveoli (Matthay et al., 2019). Therefore, injury to the epithelial and endothelial barriers of the lung is typical of ALI and directly contributes to the characteristic physiological abnormalities (Bos & Ware, 2022).

Increasing reports showing strong links between aberrant metabolism and sepsis-associated organ dysfunction (Van Wyngene, Vandewalle & Libert, 2018). In the setting of sepsis, the metabolic pathway shifts toward aerobic glycolysis to fulfill the increased ATP demand, thus contributing to the inflammatory response and tissue injury (Bar-Or et al., 2018). Experimental studies demonstrate that blockage of glycolysis is effective to improve survival outcome in sepsis and alleviates sepsis-related ALI (Yang et al., 2022; Gong et al., 2017). Additionally, higher serum lactate levels derived from glycolysis have been reported in patients with sepsis, which are positively correlated with increased mortality rates and the severity of lung injury (Vincent & Bakker, 2021; Iscra, Gullo & Biolo, 2002). Consistent with the role of lactate in inducing lactylation, septic mice manifest significant increases in global lysine lactylation and H3K14la levels in lung tissues, especially in vascular endothelial cells. Further Cut&Tag analysis reveals that H3K14la is enriched at the promoter regions of *Tfrc* and *Slc40a1*, two ferroptosis-related genes, which promotes endothelial cell ferroptosis and vascular dysfunction in sepsis-related lung injury (Gong et al., 2025). A similar effect of histone lactylation on ferroptosis is observed in AECs. High lactate-induced H3K18la at the *Mettl3* promoter site upregulates its transcription. Mettl3 increases m6A modification and stabilizes ACSL4, an essential component for ferroptosis execution. As a result, lactate accumulated during sepsis promotes ROS accumulation and mitochondria-associated ferroptosis in AECs, exacerbating lung injury in septic mice (Wu et al., 2024). PANoptosis is another form of endothelial cell death in septic lung injury. A recent experimental study reports that during sepsis, the lactylation of cold-inducible RNA-binding protein (CIRP) facilitates its release from macrophages. The extracellular CIRP is then internalized by pulmonary vascular endothelial cells, stabilizes ZEB1 expression, and amplifies ZBP1-dependent PANoptosis (Gong et al., 2024). As an endothelial gatekeeper, the pulmonary endothelial glycocalyx is a glycosaminoglycans and proteoglycans-enriched layer that covers the luminal surface of the vascular endothelium. Lactylation is also shown to enhance endothelial glycocalyx degradation and worsen ALI in mice with polymicrobial sepsis. On one hand, enriched H3K18la at the promoter of *Egr1* facilitates its transcription in pulmonary microvascular endothelial cells; on the other hand, K364 lactylation of EGR1 protein promotes its nuclear translocation. EGR1 is a pivotal upstream transcription factor of heparanase (HPSE), which mediates glycocalyx degradation and aggravates pulmonary vascular permeability (Lu et al., 2025). These findings unveil the key role of lactylation in the pathological processes of sepsis-related ALI, providing novel perspectives and potential strategies for disease treatment.

## Development of Drugs Targeting Lactylation

Lactylation has a crucial role in respiratory disease processes, spurring investigation into drugs that target this modification as a promising therapeutic approach. With numerous studies are ongoing, the current drug development targeting lactylation mainly focuses on inhibitors and compounds that affect lactate production, transport, as well as enzymatic processes underlying lactylation (Table S2).

### Targeting lactate production

Lactylation level is closely related to the concentration of lactate, which is predominantly derived from glycolytic pathway. Therefore, regulating the expression or activity of glycolytic enzymes can effectively reduce lactate production and lactylation modification. 2-DG is a glucose analog that competitively inhibits glucose metabolism, disrupting glycolysis through its effects on hexokinase (Zhu et al., 2005). Studies indicate that 2-DG treatment diminishes lactate production and histone lactylation in macrophages, alleviating crystalline silica-induced pyroptosis and lung inflammation (You et al., 2024). Similarly, 2-DG has demonstrated protective effects in sepsis-associated lung injury (Wu et al., 2024). On the basis of preclinical efficacy, a phase I dose-escalation trial of 2-DG has been executed in patients with solid tumors, including NSCLC, head and neck cancer, and breast cancer (Raez et al., 2013). Another drug, dichloroacetate (DCA), blocks pyruvate dehydrogenase kinase (PDK), enhances the entry of pyruvate into the TCA cycle, and decreases lactate production. DCA has been reported to reduce lactylation levels and modulate physiopathological processes driven by lactylation, such as angiogenesis, T cell plasticity, and cardiac repair post-myocardial infarction (Wang et al., 2023b; Wang et al., 2022). Results from a phase II clinical trial in which DCA is added to cisplatin-based chemoradiotherapy in locally-advanced head and neck squamous cell carcinoma show that this agent is safe with no detrimental effect on survival and expected metabolite changes (Powell et al., 2022), which supports further studies of metabolic drugs in the clinical setting.

LDH is a tetrameric protein composed of LDHA and LDHB, exists in five isomeric forms and catalyzes the interconversion of pyruvate and lactate. LDHA preferentially converts pyruvate to lactate, whereas LDHB mediates the reverse reaction (Valvona et al., 2016). LDHA inhibitors, including Oxamate, GSK2837808A, and (R)-GNE-140 have shown success in lowering lactylation levels in preclinical studies. Oxamate is a structural analog of pyruvate that competes with pyruvate for LDHA binding and thus suppresses LDHA activity (Kim et al., 2019). Treatment of LUAD cells with Oxamate remarkably decreases lactate production and H3K18la levels, thereby inhibiting LUAD cell malignant behaviors and tumor growth in mice (Wang et al., 2023a). GSK2837808A is a potent and selective LDHA inhibitor, and it causes significant cell cycle arrest in brain metastatic lung cancer cells by modulating cellular lactate and H3K12 lactylation (Duan et al., 2023). (R)-GNE-140, another LDHA-specific inhibitor, similarly inhibits lactate production, reduces H3K9 lactylation, and attenuates myogenesis (Dai et al., 2023). Some natural compounds can act as LDHA inhibitors. For instance, galloflavin suppresses LDHA activity, causes a decrease in lactate production and induces Burkitt lymphoma regression (Manerba et al., 2012; Vettraino et al., 2013). Salidroside also inhibits LDH activity and lowers lactate levels in muscle tissues (Liu et al., 2025b). Besides, the application of small interfering RNAs (siRNAs) to target LDHA has also been shown to be a useful means to compromise the progression of glycolysis-dependent malignancies (Wang et al., 2017; Li et al., 2016).

### Targeting lactate transport

Since lactate can be shuttled between cells through MCTs, targeting MCTs might constitute a promising approach for treating lactylation-related disorders. There are a variety of MCT inhibitors. Flavonoids including phloretin, quercetin, and α-cyano-4-hydroxycinnamate (CHC), are classical MCT1/4 inhibitors that have shown therapeutic potential for lung cancer, breast cancer, renal cell carcinoma, colorectal cancer, and osteosarcoma, et al. (Harris et al., 2009; Singh et al., 2021; Amorim et al., 2015; Miranda-Goncalves et al., 2020; Zhao et al., 2014). Several other MCT inhibitors, such as simvastatin, 4,4′-diiso-thiocyanatostilbene-2,2′-disulphonic acid (DIDS), and lonidamine, have been proven effective in various cancers (Perez-Escuredo et al., 2016; Nath et al., 2016; Izumi et al., 2011). Nevertheless, these inhibitors, particularly the initially identified phloretin, quercetin, and CHC, have limited specificity for different subtypes of MCT (Lin et al., 2022). Only recently have other highly potent and selective MCT inhibitors been developed, like AZD3965 and BAY-8002 for MCT1 (Beloueche-Babari et al., 2017; Wagner et al., 2021), AR-C15585 for MCT1/2 (Saulle et al., 2020), 7ACC2 for MCT1 and mitochondrial pyruvate transport (Corbet et al., 2018; Draoui et al., 2014), and VB124 for MCT4 (Zhang et al., 2024b). Blocking MCT1 with AZD3965 notably decreases exogenous lactate-induced H3K18la in Treg cells, downregulating the expression of TNFR2 and immunosuppressive molecules and delaying the progression of MPE (Xue et al., 2024). A phase I expansion study of AZD3965 in patients with diffuse large B-cell lymphoma and Burkitt lymphoma has been completed, and the result shows that it can be safely administered at 10 mg twice a day (NCT01791595). Moreover, CD147 augments MCT1/4 expression and acts as a chaperone for their membrane presentation (Dana et al., 2020). Therefore, targeting CD147 could also reduce intracellular lactate and lactylation levels, such as using the CD147 antibody MEM-M6/1 (Baba et al., 2008), AC-73 that disrupts CD147 dimerization (Fu et al., 2016), and p-chloromercuribenzene sulfonate (pCMBS) that affects MCT binding to CD147 (Wilson et al., 2005; Kopnick, Geistlinger & Beitz, 2021).

### Targeting lactylation processes

Lactylation not only requires lactate but is also a reversible enzymatic reaction, allowing for the modulation of enzyme activity to influence this process. Inhibiting enzyme activity of p300 by C646 reduces lactate-induced HMGB1 lactylation in macrophages, thereby improving survival outcome in polymicrobial sepsis (Yang et al., 2022). Similarly, another p300 inhibitor A485 and the KAT5 inhibitor MG149 are effective in reducing lactylation levels (Wang et al., 2023b; Jia et al., 2023). Moreover, β-alanine has recently been reported to compete with lactate for binding to AARS1, attenuate p53 lactylation, and inhibit tumorigenesis in mouse models (Zong et al., 2024). On the contrary, HDACs and SIRTs serve as established “erasers” of lactylation, and the application of the HDAC antagonists MS-275 reinforces lactylation (Dai et al., 2022), whereas the SIRT3 agonist Honokiol promotes delactylation (Jin et al., 2023).

In conclusion, targeted modulation of lactate metabolism and lactylation process sets the path for new therapeutic avenues in a range of diseases. Treatment approaches targeting glycolytic enzymes, lactate transporters, and regulatory enzymes involved in lactylation show potential for future drug development. Nevertheless, it remains challenging to optimize specificity and minimize off-target effects of these agents in the intricate disease microenvironment. Further investigation will assist in translating these findings into clinical applications.

## Concluding Remarks

Previously regarded as a waste product, lactate now shows significant involvement in diverse physiological processes. Growing evidence underscores its role as a pleiotropic signaling molecule, facilitating extensive studies into lactate-induced lactylation as a novel PTM. Lactylation process is dynamically governed by lactyltransferases and delactylase, and occurs on both histone and non-histone proteins, which modulates gene transcriptional activity and protein biological functions, respectively. Studies have shown that it is involved in the regulation of energy metabolism, angiogenesis, ferroptosis, fibrosis, immune response and barrier integrity. By influencing these processes, lactylation plays a pivotal role in disease pathogenesis, including malignancies and diseases of the lung. On the therapeutic front, interventions focusing on lactate production and transport, and lactylation-related enzymes emerge as a potential solution. Despite these overall gains, there are still some scientific questions to be answered. Firstly, although present studies concentrate on lysine residues for lactylation, it is still unknown if this modification is confined to lysine or if it can take place on other amino acids. Secondly, the currently known writer proteins of lactylation are histone acetyltransferases and alanyl-tRNA synthetases, with histone deacetylases serving as the primary erasers. However, the enzymes that dedicatedly catalyze or erase lactylation have not yet been identified. Thirdly, the complex interaction between lactylation and other forms of histone modifications, like histone acetylation, requires additional exploration. Finally, the majority of studies targeting lactylation are preclinical, utilizing cell and animal models, and do not have solid clinical evidence. Thus, additional clinical investigations are necessary to validate the effectiveness of lactylation-targeted therapies for human diseases. Addressing these questions are crucial to fully understand the functions and mechanisms of lactylation in the development of respiratory diseases, and bring new insights for refining current disease therapeutic strategies.

##  Supplemental Information

10.7717/peerj.20548/supp-1 Supplemental Information 1 Flowchart of the literature selection process

10.7717/peerj.20548/supp-2 Supplemental Information 2 Summary of studies concerning lactylation in respiratory diseases

10.7717/peerj.20548/supp-3 Supplemental Information 3 Drugs that target lactylation

## Additional Information and Declarations

### Data Availability

The following information was supplied regarding data availability: Raw data was not generated in this literature review.
