# Supplementary figures and images for: New insights into lactylation in respiratory diseases: progress and perspectives
Source: PeerJ. 2026 Jan 9;14:e20548. doi: 10.7717/peerj.20548 (PMC12794642; doi:10.7717/peerj.20548)

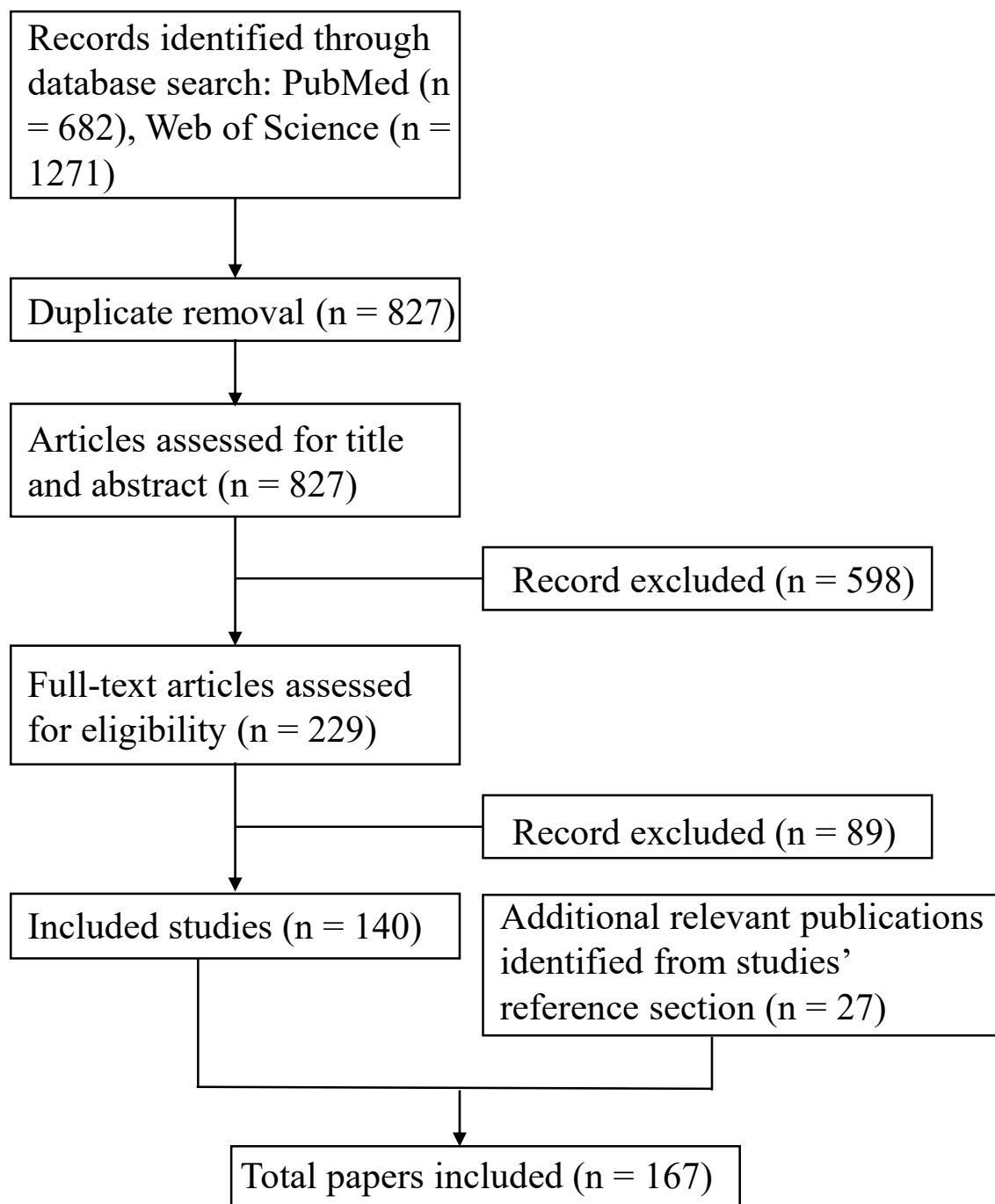

Supplement: Supplemental Information 1 [file peerj-14-20548-s001.pdf]
