# Supplementary material for: New insights into lactylation in respiratory diseases: progress and perspectives
Source: PeerJ. 2026 Jan 9;14:e20548. doi: 10.7717/peerj.20548 (PMC12794642; doi:10.7717/peerj.20548)
Supplement: Supplemental Information 2 [file peerj-14-20548-s002.docx]

**Table 1. Summary of studies concerning lactylation in respiratory diseases**

| **Diseases** | **Lactylation sites** | **Targets** | **Cell types** | **Main findings** | **Reference** |
| --- | --- | --- | --- | --- | --- |
| Asthma | / | / | Macrophage | The effect of DEX on asthma control is associated with its suppressing of Hif-1α-glycolysis-lactate axis and subsequent protein lactylation | [70] |
| Lung cancer | Histone | HK-1 and IDH3G | NSCLC cell | Lactate promotes NSCLC cell proliferation and modulates cellular metabolism through histone lactylation-mediated gene expression | [75] |
|  | H3K18 | AIM2 | Lung cancer cell | Histone lactylation of AIM2 suppresses ferroptosis and promotes lung cancer progression | [76] |
|  | H3K18 | POM121 | NSCLC cell | H3K18 lactylation potentiates NSCLC immune escape | [77] |
|  | H3K18 | IDH3G | LUAD cell | BZW2 could regulate LUAD progression through glycolysis-mediated IDH3G lactylation | [78] |
|  | H4K8 and H4K16 | Sp1 | NSCLC cell | LKB1 promotes cellular senescence and telomerase regulation through inﬂuencing the expression of TERT via histone lactylation in lung adenocarcinoma | [79] |
|  | / | SOX9 | NSCLC cell | Hypoxia facilitates the lactylation modification of SOX9 to promote cell stemness, migration, and invasion of NSCLC by enhancing glycolysis | [80] |
|  | Histone | MYCN | Lung cancer cell | The Numb/Parkin-directed mitochondrial fitness is a key metabolic switch and a promising therapeutic target on cancer cell plasticity through the regulation of histone lactylation | [84] |
|  | H4K12 | CCNB1 | Lung cancer cell | AKR1B10/glycolysis/H4K12la/CCNB1 promotes acquired PEM chemoresistance in lung cancer brain metastasis | [85] |
|  | K70 | APOC2 | Lung cancer cell | Lactyl-APOC2-K70 induces immunotherapy resistance by promoting extracellular lipolysis | [86] |
|  | H3K18 | NF-κB p65 | Treg cell | Lactate modulates gene transcription of NF-κB p65 through H3K18la, subsequently upregulating the gene expression of TNFR2 and expediting the progression of MPE | [88] |
|  | H3K18 | FOXP3 | NKT-like cell | Lactate induces FOXP3 expression in NKT-like cells in MPE | [89] |
| IPF | H3K18la | Acta2 and Col1a1 | Fibroblast | Lactate-mediated histone lactylation promotes the expression of profibrotic genes in activated lung fibroblasts | [99] |
|  | H3K18 | YTHDF1 | Alveolar epithelial cell | H3K18la promotes the progression of arsenite-related IPF via YTHDF1/m^6^A/NREP | [100] |
|  | Histone | ARG1, PDGFA, THBS1, and VEGFA | Macrophage | Lung myofibroblasts promote the profibrotic activity of macrophages via lactate-induced histone lactylation | [101] |
|  | Histone | Tgfb, Vegfa, and Pdgfa | Macrophage | PM2.5-induced glycolysis and subsequent histone lactylation in macrophages play a critical role in PM2.5-associated pulmonary fibrosis | [103] |
| Silicosis | Histone | / | Macrophage | Glycolytic reprogramming contributes to CS-induced NLRP3-dependent macrophage pyroptosis and pulmonary inﬂammation through promoting lactylation modification | [109] |
| PH | H3K18 | Bmp5, Trpc5, and Kit | PASMC | Glycolytic shift driven by mROS-mediated HIF-1α stabilization promotes hypoxic PASMC proliferation and PA remodeling | [115] |
|  | H3K18 | Gbe1, Pgf, Mt2A, Ythdf2 and Gys1 | PASMC | Hypoxia-induced upregulation of histone lactylation drives PASMC proliferation and PA remodeling | [116] |
|  | Histone | Prelamin A | PASMC | Histone lactylation-derived prelamin A accelerates PASMC senescence. Senescent PASMC secrete IL-6 to induce excessive PASMC proliferation and facilitate PA remodeling | [117] |
|  | / | FUS | PASMC | FUS lactylation results in the disruption of the redox homeostasis and causes ferroptosis in human PASMCs | [119] |
|  | H3K18 | IL-1β, IL-6, and TNF-α | PASMC | The lactylation of promoter regions within inflammatory genes leads to inflammatory phenotypic transition of PASMCs | [118] |
| ALI | / | HMGB1 | Macrophage | Lactate promotes macrophage HMGB1 lactylation and release in polymicrobial sepsis | [52] |
|  | H3K14 | TFRC and SLC40A1 | Pulmonary endothelial cell | Lactate-dependent H3K14la regulates EC ferroptosis to promote vascular dysfunction during sepsis-induced lung injury | [130] |
|  | H3K18 | METTL3 | Alveolar epithelial cell | Lactate induces ferroptosis via the GPR81/H3K18la/METTL3/ACSL4 axis in alveolar epithelial cells during sepsis-associated ALI | [131] |
|  | / | CIRP | Macrophage | Lactic acidemia promotes macrophage‑derived eCIRP release, which mediates ZBP1‑dependent PVEC PANoptosis in sepsis‑induced ALI | [132] |
|  | H3K18/K364 | EGR1 | Pulmonary microvascular endothelial cells | Lactylation of H3K18 and Egr1 promotes endothelial glycocalyx degradation in sepsis-induced ALI | [133] |

DEX, dexamethasone; PEM, pemetrexed; MPE, malignant pleural effusion; IPF, idiopathic pulmonary fibrosis; PH, pulmonary hypertension; PASMC, pulmonary artery smooth muscle cell; PA, pulmonary artery; ALI, acute lung injury; PVEC, pulmonary vascular endothelial cell
