# Supplementary material for: New insights into lactylation in respiratory diseases: progress and perspectives
Source: PeerJ. 2026 Jan 9;14:e20548. doi: 10.7717/peerj.20548 (PMC12794642; doi:10.7717/peerj.20548)
Supplement: Supplemental Information 3 [file peerj-14-20548-s003.docx]

**Table 2. Drugs that target lactylation**

| Target | Drug | Dosage | Disease model/cell type | Reference | Clinical trail |
| --- | --- | --- | --- | --- | --- |
| HK | 2-DG | 50/100/200 mg/kg | Crystalline silica-induced silicosis | [109] | Dose Escalation Trial, Phase  I/II Trial |
|  |  | 0.5 g/kg | Sepsis-associated lung injury | [131] |  |
| PDK | DCA | 200 mg/kg | Oxygen-induced retinopathy | [48] | Phase I/II Trial |
|  |  | 5 mM | BMDMs | [136] |  |
| LDHA | Oxamate | 250/500/750 mg/kg | Mouse xenograft model of lung adenocarcinoma | [78] | / |
|  | GSK2837808A | 10 µM | PC9-BrM3 cells | [85] | / |
|  | (R)-GNE-140 | 5/10 µM | C2C12 cells | [140] | / |
|  | Galloflavin | 0-500 µM | PLC/PRF/5 cells | [141] | / |
|  |  | 0-200 µM | Burkitt lymphoma cell lines and lymphoblastoid cell lines | [142] |  |
|  | Salidroside | 50 mg/kg | Overtraining-related hepatic fibrosis | [143] | Phase II Trial |
| MCT1 | AZD3965 | 50 mg/kg | Mouse xenograft model of human lymphoma | [154] | Phase I Trial |
|  |  | 2.5/5/10 mM  100 mg/kg | Treg cells  Mouse MPE models | [88] |  |
|  | BAY-8002 | 0-1 µM | HeLa cells | [155] | / |
|  | 7ACC2 | 20 µM  3 mg/kg | Tumor spheroids  Mouse xenograft model of cervix cancer | [157] | / |
|  |  | 10 µM  3 mg/kg | SiHa, HL-60, and MDA-MB231 cells  Mouse xenograft models | [158] |  |
| MCT1/2 | AR-C15585 | 1/2.5/5/10 µM | Leukemic cell lines | [156] | / |
| MCT4 | VB124 | 10 µM  30 mg/kg | Foam cells  HFD-fed ApoeKO mice | [159] | / |
| p300 | C646 | 5 µM | RAW 264.7 cells | [52] | / |
|  | A485 | 200 μM | Oxygen-induced retinopathy | [48] | / |
| KAT5 | MG149 | 20 μM | HEK293T cells | [55] | / |
| AARS1 | β-alanine | 1.2% in drinking water (w/v) | Mouse breast cancer model | [165] | / |
| HDAC | MS-275 | 1 µM | P19 EC cells | [166] | Phase I/II/III Trial |
| SIRT3 | Honokiol | 10 mg/kg | Mouse HCC model | [167] | Phase I/III Trial |
| CD147 | AC-73 | 5/10 µM  25/50 mg/kg | HCC cell lines  Orthotopic transplant nudemouse model of HCC metastasis | [162] | / |
|  | pCMBS | 0.5 mM | Rabbit erythrocytes | [163] | / |
|  |  | 30 µM | Yeast cells | [164] |  |

DCA, dichloroacetate; HFD, high-fat diet; MPE, malignant pleural effusion; HCC, hepatocellular carcinoma; pCMBS, pchloromercuribenzene sulfonate
